# Are endemics functionally distinct? Leaf traits of native and exotic woody species in a New Zealand forest

**DOI:** 10.1371/journal.pone.0196746

**Published:** 2018-05-02

**Authors:** J. Mason Heberling, Norman W. H. Mason

**Affiliations:** 1 Section of Botany, Carnegie Museum of Natural History, Pittsburgh, PA, United States of America; 2 Department of Ecology and Evolutionary Biology, The University of Tennessee, Knoxville, TN, United States of America; 3 Department of Biology, Syracuse University, Syracuse, NY, United States of America; 4 Landcare Research, Hamilton, New Zealand; Chinese Academy of Forestry, CHINA

## Abstract

Recent studies have concluded that native and invasive species share a common set of trait relationships. However, native species in isolated regions might be functionally constrained by their unique evolutionary histories such that they follow different carbon capture strategies than introduced species. We compared leaf traits relating to resource investment, carbon return, and resource-use efficiency in 16 native (endemic) and three non-native (invasive) species in a temperate forest in Canterbury, South Island, New Zealand. Trait differences were more closely associated with leaf habit than nativity. Deciduous species (including invaders) exhibited greater maximum photosynthetic rates at similar resource costs, which resulted in greater nitrogen- and energy-use efficiencies than evergreen natives. Leaf area was the only trait that differed significantly by nativity (over two-fold larger in invaders). Invaders and deciduous natives both occupied the ‘fast return’ end of the leaf economics spectrum in contrast to the native evergreens which had comparatively slow return on investment. Dominant woody invaders in this forest are physiologically distinct from many New Zealand endemic species, which are overwhelmingly evergreen. It remains unclear whether these trait differences translate to an ecological divergence in plant strategy, but these results suggest that ecophysiological tradeoffs are likely constrained by biogeography.

## Introduction

It is often assumed that the success of invasive plant species arises from functional differences relative to native species, which provide physiological advantages in particular ecological contexts [1], especially in habitats with high levels of disturbance or resources [2,3]. A leading framework for understanding leaf trait variation has been the ‘worldwide leaf economic spectrum’ (LES;[4]), which describes coordinated leaf trait cost-benefit tradeoffs across biomes and life forms. The LES runs from species that have a quick return in leaf investment (i.e., productive strategies typified by traits such as high specific leaf area, short leaf lifespan, high photosynthetic capacity, high nutrient content) to contrasting trait strategies that favor resource conservation and tissue persistence. LES theory suggests that all species share similar carbon capture strategies, as assessed through common slopes and y-intercepts in important trait relationships. Trait combinations (strategies) outside of this general LES are thought to be either subject to strong directional selection (ecologically constrained) or genetically/biophysically impossible [5]. Recently, these generalizations have led to debate both within invasion-centered research (e.g., [6–8]) as well as the functional trait literature at large (e.g., [9,10]. On one hand, many authors have concluded that invasive and native species are not ‘fundamentally’ different—that is, bivariate trait scalings (slopes) between traits linked to resource investment and rate of return are quantitatively the same, indicating common carbon capture strategies (e.g., [6,11]). On the other hand, a growing number of native-invasive comparisons have reported deviations from core trait relationships and/or resource-use efficiency differences in invasive species (e.g., [8,9,12–16]). These studies suggest different physiological constraints on resource capture and use, as evinced by differences in bivariate trait relationships (e.g., slope, y-intercept) or carbon gain per unit resource cost (resource use efficiency). Results are mixed or equivocal for the adaptive value of deviations from a theoretical universal tradeoff surface, and further, what would truly constitute a biologically significant deviation. It is increasingly clear that the strength and interpretation of resource-use patterns differ depending on community context [17]. Therefore, results from global comparisons of invasive and native plant species are not necessarily repeated at smaller scales [8].

Botanists have long recognized vast diversity in the size, age, geological history, and phylogenetic richness of the word’s regional floras. In particular, island ecosystems have traditionally been a focus of invasion case studies [18], citing their reduced biotic resistance due to fewer resident species, absent life forms, or the evolution of less competitive phenotypes compared to those on the mainland. Extending this general idea, Fridley & Sax [19] proposed the ‘Evolutionary Imbalance Hypothesis’ that predicts successful invaders originate from areas in regions with large populations, long periods of time under stable conditions and relatively high selection pressures with strong competition. Considering the converse, floras that evolved under the opposite conditions (i.e., small regions with high geographic isolation, historically unstable environmental conditions and low competition) should include species which have evolved intrinsically suboptimal strategies for resource-use, competition, and reproduction. Therefore, the nature of trait tradeoffs (e.g., slopes, y-intercepts) may differ. Further, in the context of invasions, reduced herbivore pressure on non-natives in the introduced range could change the metabolic costs such that they are less constrained to a particular combination of trait values (enemy release hypothesis;[20]). Hypothesized strategy differences in plants that evolved in different regions has preliminary support from biogeographic analyses of leaf trait relationships [21], case studies of novel invaders into phylogenetic constrained communities [22], and the non-random global exchanges of non-native plants [19,23].

The New Zealand flora is one of the most isolated and evolutionarily distinct in the world, marked by a high degree of species endemism. The distinct visual appearance of New Zealand vegetation (e.g., divaricating architecture with interlacing wide-angled branching; strikingly different phenotypes between juveniles and adults) characterizes the uniqueness of the flora [24]. For many species, the adaptive significance of such traits has yet to be fully understood. In a biogeographic analysis of New Zealand tree species, McGlone *et al*. [25] found significant differences in species richness and plant traits compared to similar temperate tree floras. Further, less than 5% of New Zealand species are deciduous (broadly defined as pronounced leaf loss in winter), hypothesized to be due to phylogenetic constraint, but also strongly due to present day climate, particularly relatively mild winters [26]. In this biogeographic context, New Zealand has many “functional gaps” in the regional flora [27].

It is not surprising that many ecologists have questioned whether the traits of New Zealand native species are best adapted for present-day environmental conditions [24,26,27]. In light of potential functional gaps in the regional flora, New Zealand might be especially vulnerable to invasion by functionally novel non-native species [28]. Studies have explored the functional ecology of the native species (e.g., [29,30]), as well as the significance of peculiar endemic traits [31,32]. A recent demographic study has shown a novel divergence in the classic growth vs. shade tolerance tradeoff among native species [33]. Comparative trait studies between native and invasive species in New Zealand have found important similarities and differences in growth and carbon gain strategies [34,35]. However, it remains unknown whether invasions in New Zealand can be partly explained by unique strategies evolved in the endemic flora, or alternatively, if endemics and invaders follow the same ecophysiological tradeoffs generalized for species worldwide (e.g., [4]). Due to different selection pressures (e.g., reduced competition, herbivory), it is possible that endemics have evolved functionally unique strategies compared to other species, such as contrasting slopes in common cost-benefit tradeoffs or exhibiting novel combination of trait values.

In a New Zealand forest, we measured leaf-level photosynthetic traits, energy and nitrogen investments, and resource-use efficiencies of co-occurring endemic (native) and invasive (non-native) woody species. We asked whether modern plant invasions in historically isolated regions can be understood as deviations from traditional plant strategy tradeoffs. Due to their unique evolutionary history, we hypothesized that New Zealand endemics would exhibit significant differences from invaders in their plant strategies—that is, contrasting slopes in their trait relationships and occupying novel trait space (*sensu* [6,8]). Further, since native deciduous species do not dominate any vegetation type in New Zealand [26], we also hypothesized that invaders, all of which were deciduous, would functionally differ from deciduous endemics and exhibit trait syndromes associated with greater productivity and resource acquisition.

## Materials and methods

### Study site and sampling protocol

Peel Forest Park Scenic Reserve is a remnant Podocarpaceae forest located along the foothills of the Southern Alps in the Canterbury Region, South Island, New Zealand (43°53′ S, 171°14′ E). Mean temperature and annual precipitation are 10.5°C and 1065 mm, respectively. The native overstory primarily consists of Podocarpaceae members *Dacrycarpus dacridioides* (kahikatea) and *Podocarpus totara* (totara) and the Malvaceous trees *Hoheria angustifolia* (narrow-leaved lacebark) and *Plagianthus regius* (ribbonwood), along with a rich sub-canopy and early successional species typical of nutrient-rich habitats in the region. Patches of mature, remnant, and successional forest occur within a larger matrix of grassland, regenerating shrubland, and thickets dominated by *Rubus fruticosus* (European blackberry). The forest understory, forest edge, and open habitats are invaded by a variety of woody species, primarily of European origin. In certain areas, the overstory canopy is dominated by the invaders *Acer pseudoplatanus* (sycamore maple) and *Fraxinus excelsior* (European ash). Sampling permissions were obtained from The Department of Conservation, New Zealand.

To encompass the diversity of trait syndromes of co-occurring species in this forest, we targeted 16 broadleaved native species and 3 invasive species (Table 1). Our sample size was limited in the invasive comparison, as only three invasive species were abundant in this forest. As much as possible, individuals of each species were equally sampled across forest edge, gap, and closed canopy understory conditions to capture intraspecific variation at this site. From early-mid December 2013 (austral summer), four to fourteen individuals per species were sampled (mean ± SD: 7 ± 3 individuals per species; Table 1). Gas exchange measurements were performed on cut branches, following the protocol of [36], widely used for temperate woody species (e.g., [9]). We used cut branches for logistical reasons (access to sites) and to ensure measurements were made under consistent environmental conditions. At least two upper branches per individual were cut in the field on cool, damp mornings, and immediately recut under water. To maintain xylem water potential, the severed ends were wrapped with wet paper towel, placed in plastic bags, and stored in a cooler to minimize transpiration until transported to the lab. Upon returning to lab, branches were recut and cut stems placed in water, loosely covered in transparent plastic, and stabilized at room temperature under low light for 1–3 d before recording gas exchange measurements. Each morning, branches were recut under fresh water. There was no indication of time effect on gas exchange during the measurement period. Protocol tests on other woody species suggest most species exhibit consistent light saturated photosynthetic rates immediately following branch cutting, but stomatal conductance decline during longer measurement periods [36].

**Table 1:** Woody species measured, including invasive status, biogeographic origin, growth form, and number of replicate individuals.

Family	Species	Code	Invasive status	Origin	Leaf Habit^‡^	Growth Form	n^¶^
Adoxaceae	*Sambucus nigra* L.	SamNig	invasive	European	deciduous	shrub	5 (2)
Araliaceae	*Pseudopanax arboreus* (L.f.) Philipson	PseArb	-	NZ endemic	evergreen	tree	4 (4)
	*Schefflera digitata* J.R. Forst. & G.Forst	SchDig	-	NZ endemic	evergreen	tree	10 (7)
Elaeocarpaceae	*Aristotelia serrata* (J.R. Forst. & G.Forst) Oliv.	AriSer	-	NZ endemic	semi-deciduous	tree	8 (6)
	*Elaeocarpus hookerianus* Raoul	ElaHoo	-	NZ endemic	evergreen	tree	5 (2)
Fabaceae	*Sophora microphylla* Aiton	SopMic	-	NZ endemic	evergreen*	tree	4 (4)
Griseliniaceae	*Griselinia littoralis* (Raoul) Raoul	GriLit	-	NZ endemic	evergreen	tree	5 (5)
Malvaceae	*Hoheria angustifolia* Raoul	HohAng	-	NZ endemic	evergreen*	tree	7 (0)
	*Plagianthus regius* (Poit.) Hochr.	PlaReg	-	NZ endemic	deciduous	tree	6 (6)
Oleaceae	*Fraxinus excelsior* L.	FraExc	invasive	European	deciduous	tree	8 (5)
Onagraceae	*Fuchsia excorticata* (G.Forst.) L.f.	FucExc	-	NZ endemic	deciduous	tree	4 (4)
Pennantiaceae	*Pennantia corymbosa* J.R. Forst. & G.Forst	PenCor	-	NZ endemic	evergreen	tree	5 (3)
Pittosporaceae	*Pittosporum eugenioides* A.Cunn.	PitEug	-	NZ endemic	evergreen	tree	10 (8)
	*Pittosporum tenuifolium* Sol. Ex Gaertn.	PitTen	-	NZ endemic	evergreen	tree	5 (5)
Rubiaceae	*Coprosma rotundifolia* A.Cunn.	CopRot	-	NZ endemic	evergreen	shrub	9 (7)
Rutaceae	*Melicope simplex* A.Cunn.	MelSim	-	NZ endemic	evergreen	shrub	7 (3)
Sapindaceae	*Acer pseudoplatanus* L.	AcePse	invasive	European	deciduous	tree	14 (10)
Violaceae	*Melicytus ramiflorus* J.R. Forst. & G.Forst	MelRam	-	NZ endemic	evergreen	tree	4 (4)
Winteraceae	*Pseudowintera colorata* (Raoul) Dandy	PseCol	-	NZ endemic	evergreen	tree	10 (9)

^‡^Leaf habit for NZ species categorized per McGlone *et al*. (2004).

*Species with partial winter leaf loss in the outer canopy were categorized as evergreen (*S*. *microphylla*, *H*. *angustifolia*).

^**¶**^Number of replicates with complete light response curve data given in parentheses.

### Leaf gas exchange

Gas exchange measurements were made on recently expanded, mature leaves using an LI-6400 portable photosynthesis system equipped with CO_2_ and temperature control modules, 2x3 cm sample chamber and a red-blue LED light source (Li-Cor, Lincoln, NE, USA). Leaf temperature was maintained at 25°C under ambient humidity throughout measurements with sample chamber flow rate of 500 μmol s^-1^ and sample chamber CO_2_ concentration at 380 μmol mol^-1^. For leaves that did not entirely fill the chamber, leaf area was estimated using leaf scans. To improve measurement accuracy for very small-leaved species, multiple leaves were placed in the sample chamber. Leaves were photoinduced at a moderate irradiance level (300 μmol photons m^-2^ s^-1^) until equilibration prior to measuring light response curves. Irradiance levels (photosynthetic photon flux density; PPFD) were then progressively increased until light saturation (800–1,500 μmol photons m^-2^ s^-1^). All individuals were light saturated at the highest light levels, with no apparent signs of photoinhibition. After achieving light saturation, photosynthetic responses to light (A/*q* curve) were measured at 12 steps (800, 1000, 1300, 1000, 800, 500, 300, 200, 100, 50, 20, 0 μmol photons m^-2^ s^-1^). Net photosynthetic rates were recorded after equilibrating for at least two minutes at each PPFD and reaching predefined stability parameters based on photosynthetic rate and stomatal conductance.

### Leaf structural and biochemical characteristics

After photosynthetic measurements, leaf thickness was measured using a digital micrometer, taking the mean of three measurements per leaf (avoiding midrib and major leaf veins; 4 leaves per individual). Leaf chlorophyll concentration (chl) was estimated with a chlorophyll meter (atLEAF+, FT GREEN LLC, Wilmington, DE USA), using the mean of 3 readings per leaf (avoiding midrib; 4 leaves per individual). The atLEAF+ measures leaf absorptance difference between 660 nm and 940 nm and has been shown perform similarly to other readers and correlated to total chl content [37]. Leaves were then oven dried at 60°C for at least 48 hours. Specific leaf area (cm^2^ g^-1^) was calculated as the leaf surface area per g dry mass. Ground leaf samples were placed in an ashing furnace at 500°C for 4 hours, and leaf ash concentration was calculated as ash mass divided by sample mass. Duplicate samples were averaged for each individual. Leaf nitrogen (N) and carbon (C) concentrations were determined using an elemental analyzer (CE Elantech, Lakewood, NJ, USA).

Leaf construction cost (CC) quantifies the amount of glucose equivalents required to construct a leaf in terms of carbon skeletons, reductant, and ATP, excluding additional costs for maintenance and substrate transport. Leaf CC_mass_ (g glucose g^-1^) was estimated using the following equation [38,39]:

$${\mathrm{CC}}_{\mathrm{mass}}=(-1.041+5.077C_{\mathrm{mass}})(1-0.67\mathrm{Ash})+5.325N_{\mathrm{mass}}$$ (Eq 1)

where C_mass_ is leaf carbon concentration, Ash is leaf ash concentration (proxy for mineral concentration), and N_mas*s*_ is leaf nitrogen concentration (all in mg g^-1^). We assumed leaf NO_3_^-^ accumulation is negligible compared to organic N forms, and nitrate is the dominant form of N uptake. The first part of the CC equation above takes into account the carbon costs (empirically determined from the relationship between glucose costs and C content of biochemical compounds; [38]). The second part of the first term (including *Ash*) subtracts the mineral component in organic tissue from C cost, as the mineral fraction in organic tissue does not require C skeletons and energy required for their uptake is independent of costs for growth [39]. The last term of the CC equation above accounts for the additional, substantial costs required to reduce nitrate into organic N (proteins).

### Data analysis

Photosynthetic response to light (A/*q*) was modeled using a four-parameter non-rectangular hyperbola [40]:

$$A_{net}=\frac{\phi PPFD+A_{max}-\sqrt{{\left(\phi PPFD+A_{max}\right)}^{2}-4ϴ\phi PPFD(A_{max})}}{2ϴ}-R_{d}$$ (Eq 2)

where A_net_ and A_max_ are the area-based net and maximum gross photosynthetic rates (μmol CO_2_ m^-2^ s^-1^), respectively, ϕ is the apparent quantum yield (mol CO_2_ mol photons^-1^), R_d_ is daytime dark respiration rate (|A_net_| at no light; μmol CO_2_ m^-2^ s^-1^), and θ is curve convexity (dimensionless). Light compensation point (LCP) was estimated from the x-axis intercept.

The A/*q* model (Eq 2) was implemented in a hierarchical Bayesian (HB) framework with random effects to allow for species and individual level variation for each parameter (see Supporting Information S1 File). Uninformative prior distributions were used for all parameters, with certain distributions truncated to ensure sampling remained in biologically realistic trait space (S1 Table). The model included species-level random effects and the 95% Bayesian credible intervals were used to compare parameters across species and groups. We also ran models which included individual-level random effects to examine intraspecific variation in trait relationships between groups. To test for univariate differences by nativity (native or invasive) and leaf habit (deciduous or evergreen), each empirical trait (e.g., SLA) or parameter posterior distribution from the A/*q* model (e.g., A_max_) was analyzed in a HB framework as a function of nativity and leaf habit, with species and individual random effects. Analyses were performed in JAGS [41] using R2jags [42] in R [43] (see S2 File for R code). Final models were run with three parallel Markov chain Monte Carlo (MCMC) chains for 100,000 iterations, discarding the initial 50,000 for burn-in. Trace plots and the Gelman-Rubin diagnostic were used to confirm convergence.

Bivariate trait relationships were analyzed with standardized major axis (SMA) line fitting implemented with the *smatr* package in R [44] using empirical measurements or posterior means from light response curve models described above (S2 Table). Testing in the SMA routine involves first testing for common slopes between groups. If the slopes do not differ, the lines fitted to the groups may represent a shift along their common slope and/or shifts in elevation (y-intercept). When necessary, measurements were converted between area (i.e., m^-2^ leaf) and mass-based estimates (i.e., g^-1^ leaf) through their corresponding SLA.

## Results

### Trait comparisons between invasive and native species

**Fig 1:**
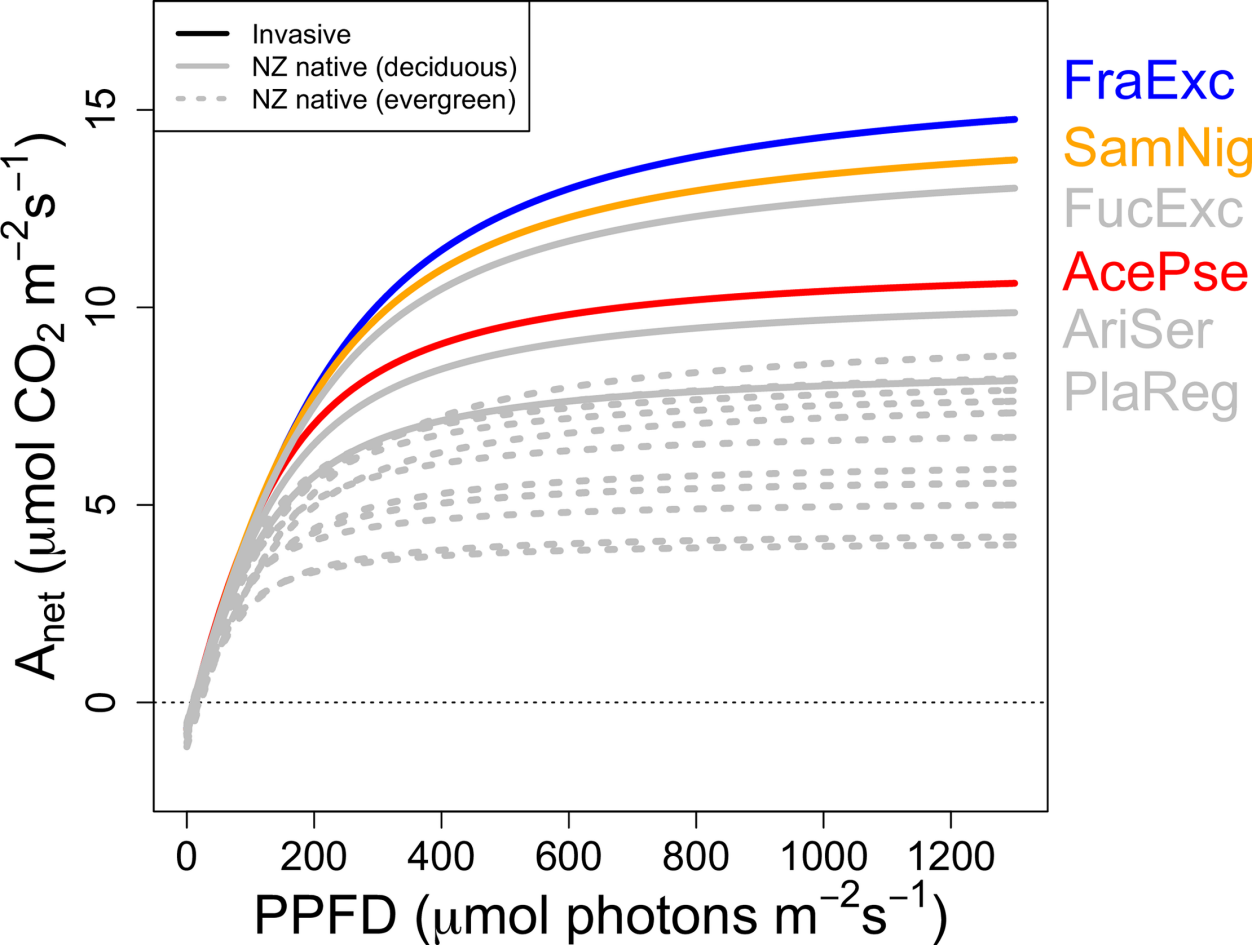
Species-level average modeled light response curves for 3 invasive (non-grey) and 15 native (grey) species. Curves estimate each species’ area-based net photosynthetic rates (A_net_) response to irradiance (photosynthetic photon flux density, PPFD), using all data with random effects for species. Only deciduous species are labeled, following codes listed in Table 1. Corresponding parameter estimates for each species are illustrated in Fig 2.

**Fig 2:**
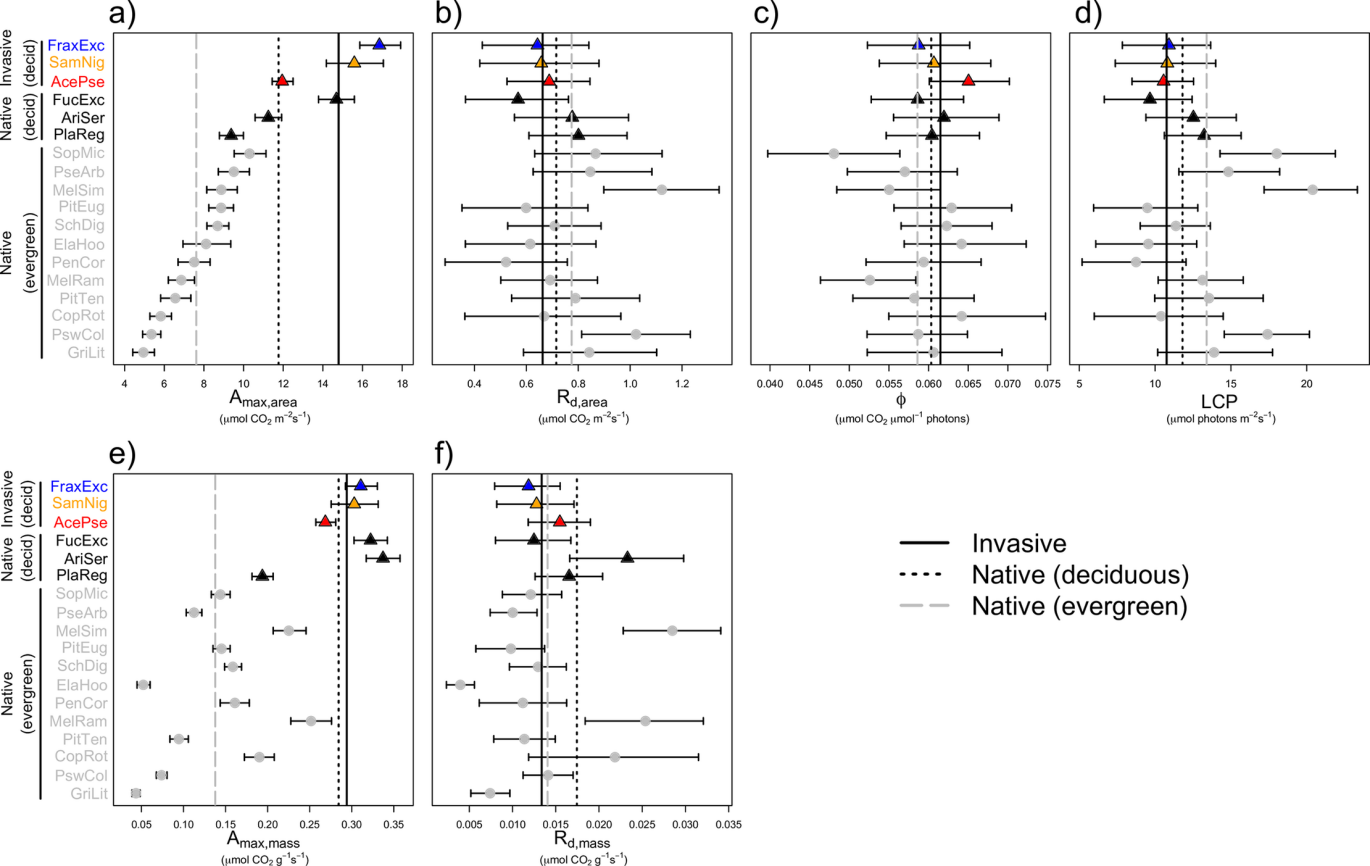
Means and 95% credible intervals by species grouped by nativity and leaf habit. (a) area-based maximum photosynthetic rate (A_max,area_), (b) area-based dark respiration rate (R_d,area_), (c) apparent quantum yield (ϕ), (d) light compensation point (LCP), (e) mass-based maximum photosynthetic rate (A_max,mass_), and (f) mass-based dark respiration rate (R_d,mass_). Vertical lines show group level averages.

Photosynthetic rates (A_net_) did not differ between native or invasive species when all natives were grouped together. However, evergreen species (all native, n = 12) had significantly lower maximum photosynthetic capacities than deciduous species (3 native, 3 invasive; Fig 1), both on a per area (43% lower A_max,area_, Fig 2A; Table 2) and a per mass basis (52% lower A_max,mass_; Fig 2E). No other photosynthetic light response curve parameters (R_d_, LCP, ϕ) significantly differed between species (Fig 2, Table 2). A_net_ was similar for all species at low and zero PPFD (R_d_, Table 2), but significantly greater in deciduous species at moderate (ca. 250 μmol photons m^-2^ s^-1^) and saturating irradiances (Fig 1).

**Table 2:** Mean values (± 1 SE) of photosynthetic, biochemical, structural, and resource-use efficiency leaf traits among native and invasive species.

Trait (units)^a^	Native (NZ endemic)			Invasive	ß_nativity_ 95% CI	ß_leaf habit_ 95% CI
	*all natives*	*evergreen*	*deciduous*	*deciduous*		
A_max,area_ (μmol CO_2_ m^-2^ s^-1^)	8.4 ± 0.6	7.62 ± 0.5	11.8 ± 1.6	14.8 ± 1.5	(-5.4, 1.5)	**(-6.9, -1.6)**
A_max,mass_ (nmol CO_2_ g^-1^ s^-1^)	167 ± 23	138 ± 19	284 ± 46	294 ± 13	(-0.6, 0.6)	**(-1.3, -0.4)**
R_d,area_ (μmol CO_2_ m^-2^ s^-1^)	0.76 ± 0.04	0.77 ± 0.05	0.72 ± 0.07	0.66 ± 0.01	(-0.09, 0.26)	(-0.18, 0.09)
R_d,mass_ (nmol CO_2_ g^-1^ s^-1^)	14.7 ± 1.8	14.1 ± 2.1	17.4 ± 3.2	13.3 ± 1.1	(-0.01, 0.01)	(-0.01, 0.001)
A_max_/R_d_	12 ± 1	11 ± 1	18 ± 5	23 ± 13	(-0.88, 0.34)	(-0.85, 0.01)
ϕ (μmol CO_2_ μmol^-1^ photons)	0.059 ± 0.001	0.059 ± 0.001	0.060 ± 0.001	0.061 ± 0.002	(-0.01, 0.01)	(-0.01, 0.01)
LCP (μmol photons m^-2^ s^-1^)	13.1 ± 0.9	13.4 ± 1.1	11.8 ± 1.1	10.8 ± 0.1	(-3.4, 4.5)	(-3.0, 2.8)
SLA (cm^2^ g^-1^)	184 ± 5	165 ± 5	251± 12	205± 9	(-0.37, 0.79)	(-0.819, 0.10)
LDMC (g g^-1^)	0.27 ± 0.02	0.28 ± 0.02	0.22± 0.02	0.26± 0.02	(-0.26, 0.12)	(-0.05, 0.26)
Leaf thickness (mm)	0.243 ± 0.005	0.252 ± 0.005	0.201 ± 0.007	0.187 ± 0.005	(-0.05, 0.15)	(-0.12, 0.18)
Leaf area (cm^2^)	60 ± 5	64 ± 6	43 ± 4	145 ± 6	**(-2.76, -0.14)**	(-1.86, 0.22)
C_mass_ (%)	48.43± 0.65	48.86 ± 0.76	46.67± 0.50	47.58± 0.65	(-3.37, 1.513)	(-0.035, 3.884)
C_area_ (g m^-2^)	33.45 ± 4.59	36.35 ± 5.43	21.86± 2.33	27.14± 1.93	(-32.29, 22.82)	(-6.719, 34.835
N_mass_ (%)	2.64 ± 0.21	2.51 ± 0.25	3.16± 0.08	3.41± 0.29	(-1.79, 1.20)	(-1.90, 0.38)
N_area_ (g m^-2^)	1.58 ± 0.13	1.61 ± 0.16	1.46± 0.20	1.89± 0.15	(-1.25, 0.45)	(-0.47, 0.82)
Chl index	46 ± 2	48 ± 3	39± 4	47± 5	(-23.80, 8.17)	(-3.78, 20.73)
Ash (mg g^-1^)	67 ± 6	64 ± 7	79± 11	73± 8	(-0.77, 0.861)	(-0.90, 0.34)
CC_mass_ (eq. g glucose g^-1^)	1.50 ± 0.03	1.51 ± 0.04	1.43± 0.03	1.49± 0.02	(-0.18, 0.06)	(-0.025, 0.171)
CC_area_ (eq. g glucose m^-2^)	103.8 ± 14.4	113.1 ± 17.0	66.8± 6.5	85.0± 6.1	(-97.73, 68.13)	(-23.13, 108.8)
PNUE (μmol CO_2_ g^-1^ N s^-1^)	5.71 ± 0.56	5.03 ± 0.41	8.46 ± 1.62	7.79 ± 0.26	(-2.44, 3.53)	**(-5.54, -0.75)**
PEUE (μmol CO_2_ kg^-1^ glucose s^-1^)	115 ± 17	94 ± 14	198 ± 29	198 ± 11	(-1.01, 1.02)	**(-1.68, -0.08)**
WUE (μmol CO_2_ mmol^-1^ H_2_O)	6.41 ± 0.81	6.72 ± 0.99	5.16 ± 0.29	6.40 ± 0.69	(-2.51, 2.19)	(-1.16, 2.46)

Native species are further separated by leaf habit. Statistical differences were assessed from the credible intervals (CI) for fixed effect coefficients for native status (ß_nativity_; native or invasive) and leaf habit (ß_leaf habit_; evergreen or deciduous) from HB models. 95% CIs that do not include zero (significant) are highlighted in bold. Evergreen species included those which may be considered briefly deciduous, with outer canopy winter leaf loss (*S*. *microphylla*, *H*. *angustifolia*).

^a^ A_max,area_ and A_max,mass_, area- and mass-based light saturated gross photosynthetic rates (A_max,mass_ = A_max,area_ x SLA)

R_d,area_ and R_d,mass_, area- and mass-based dark respiration rate; A_max_/R_d_, respiration efficiency; ϕ, apparent quantum yield; LCP, light compensation point; SLA, specific leaf area; SLA, specific leaf area; LDMC, leaf dry matter content; Thickness, leaf thickness excluding major veins; Leaf area, including all leaflets of compound leaves; C_mass_ and C_area_, mass- and area-based leaf carbon concentration; N_mass_ and N_area_, mass- and area-based leaf nitrogen concentration; Chl index, chlorophyll concentration proxy from handheld meter; Ash, leaf ash concentration; CC_mass_ and CC_area_, mass- and area-based leaf construction costs; PNUE, potential photosynthetic nitrogen-use efficiency; PEUE, photosynthetic energy-use efficiency; WUE, water-use efficiency

**Fig 3:**
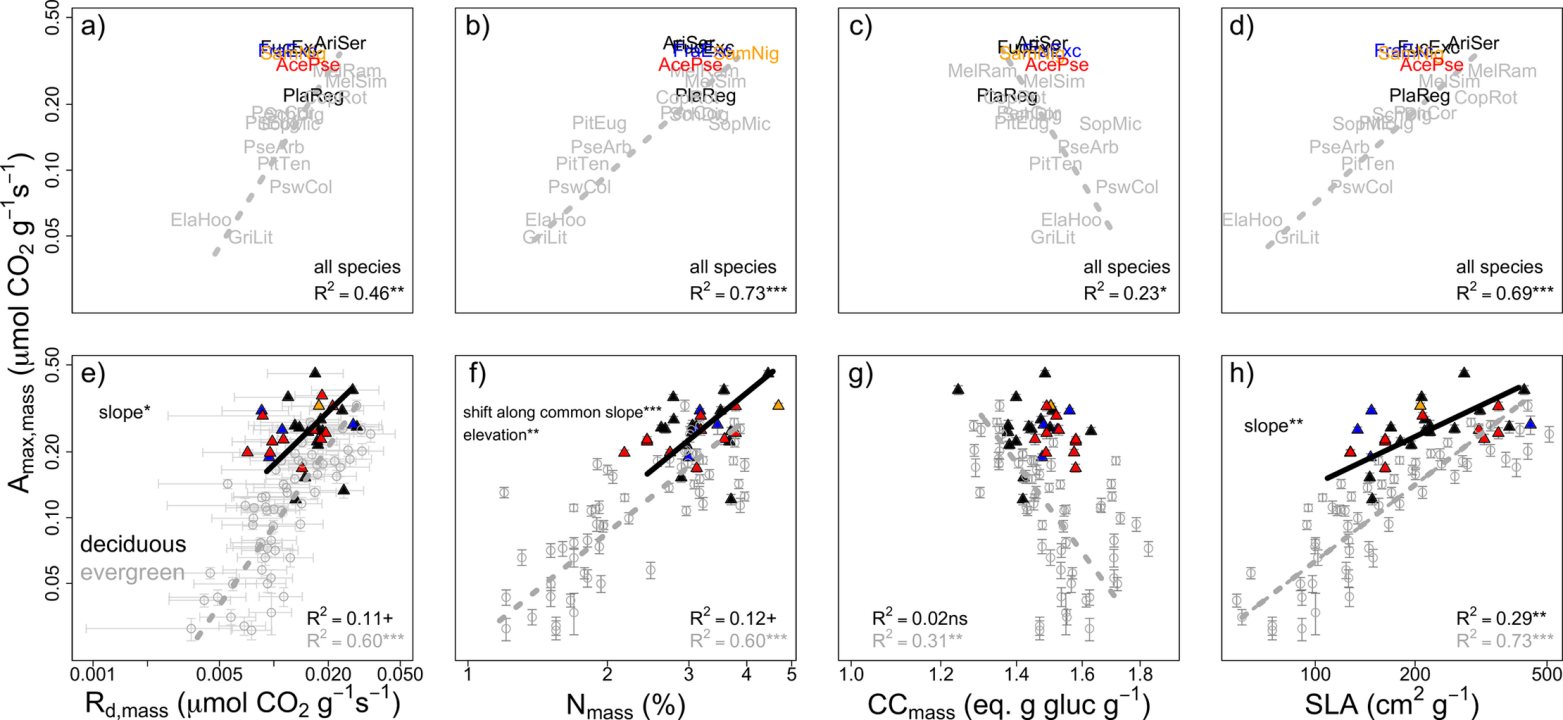
**Standardized major axis (SMA) relationships for mass-based light-saturated maximum photosynthetic rate (A**_**max,mass**_**) and leaf resource cost traits (a, e) dark respiration rate (R**_**d,mass**_**), (b, f) nitrogen concentration (N**_**mass**_**), (c, g) construction cost (CC**_**mass**_**), and (d, h) specific leaf area (SLA).** Points refer to species- (a-d) or individual-level (e-h) estimates. Native deciduous species are denoted by black text and open triangles, invasive species by closed triangles, and native evergreen species (open circles) are shown in grey. Light gray error bars denote 95% credible intervals on posterior means from light response curve models. Only significant SMA lines are drawn (deciduous, solid black line; evergreen, dashed grey line). SMA analyses were performed only for relationships showing at least moderate correlation (R^2^>0.1, *P*<0.1). Significance tests indicate differences in slope, elevation (y-intercept), or shift along common slope. +*P*<0.1; * *P*<0.05; **P<0.01; ***P<0.001. Note axes are on log scale.

Traits varied substantially both within and among species; for example, SLA and A_max,mass_ varied over tenfold and N_mass_ varied nearly fourfold across all species (Fig 3). Species means differed more by leaf habit than nativity, with invaders tending to only have slightly more extreme values than deciduous natives (Table 2). Leaf area was the only trait that differed as a function of nativity (Table 2), with invaders as a group having 2.4 times larger leaves than native on average. Similar to native deciduous species, invaders had higher mean SLA, lower leaf thickness, and lower leaf dry matter content (LDMC) than native evergreens, but none of these mean differences were statistically significant.

In terms of nutrient and energy investments, neither leaf N (N_mass_, N_area_) nor construction costs (CC_mass_, CC_area_) differed significantly by leaf habit or nativity. Mean N_mass_ was higher in deciduous species, but this difference was not significant. Due to greater A_max_ at similar resource costs (leaf N, C, CC), deciduous species (including both native and invasive) had significantly greater photosynthetic nitrogen use efficiency (PNUE = A_max_/N) and energy use efficiency (PEUE = A_max_/CC; Table 2). These efficiency differences were substantial, with 62% greater mean PNUE and 111% greater mean PEUE in deciduous compared to evergreen species.

### Bivariate tradeoffs in deciduous and evergreen species

**Fig 4:**
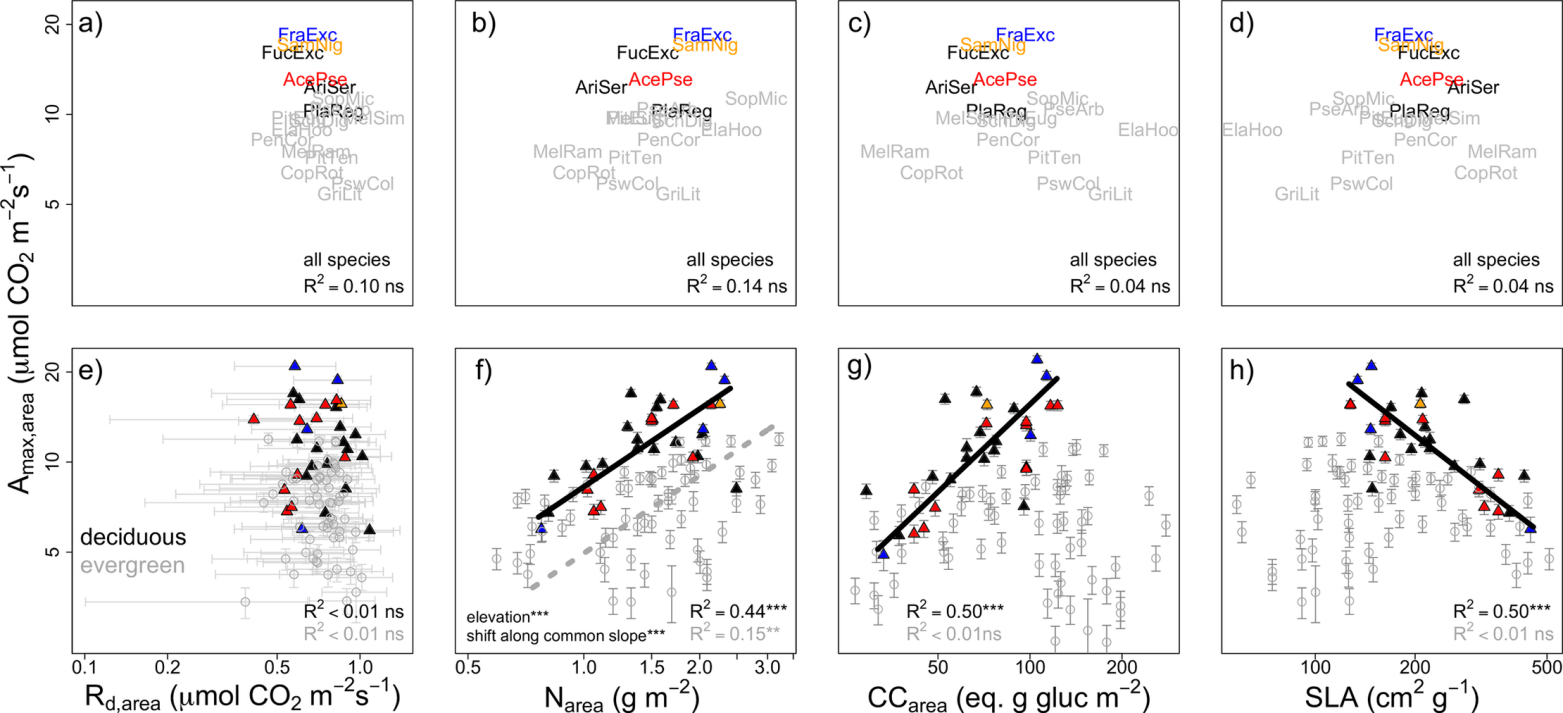
**Standardized major axis (SMA) relationships for area-based light-saturated maximum photosynthetic rate (A**_**max,area**_**) and leaf resource cost traits (a, e) dark respiration rate (R**_**d,area**_**), (b, f) nitrogen concentration (N**_**area**_**), (c, g) construction cost (CC**_**area**_**), and (d, h) specific leaf area (SLA).** Points refer to species- (a-d) or individual-level (e-h) estimates. Native deciduous species are denoted by black text and open triangles, invasive species by closed triangles, and native evergreen species (open circles) are shown in grey. Light gray error bars denote 95% credible intervals on posterior means from light response curve models. Only significant SMA lines are drawn (deciduous, solid black line; evergreen, dashed grey line). SMA analyses were performed only for relationships showing at least moderate correlation (R^2^>0.1, *P*<0.1). Significance tests indicate differences in slope, elevation (y-intercept), or shift along common slope. +*P*<0.1; * *P*<0.05; **P<0.01; ***P<0.001. Note axes are on log scale.

Considering bivariate cost-benefit trait relationships in photosynthetic capacity (A_max_) and associated leaf resource investments, invasive species clustered towards strategies characterized by high photosynthetic returns and high resource needs. As with univariate analyses, deciduous natives followed a similar pattern to those of invasive species. At the level of species means, carbon gain (A_max_)- resource cost (e.g., R_d_, N, CC, SLA) relationships were tightly correlated on a mass-basis (Fig 3; R^2^ range: 23%-73%, all *P*<0.05), but not correlated when analyzed on an area-basis (Fig 4; R^2^ ≤10%).

**Fig 5:**
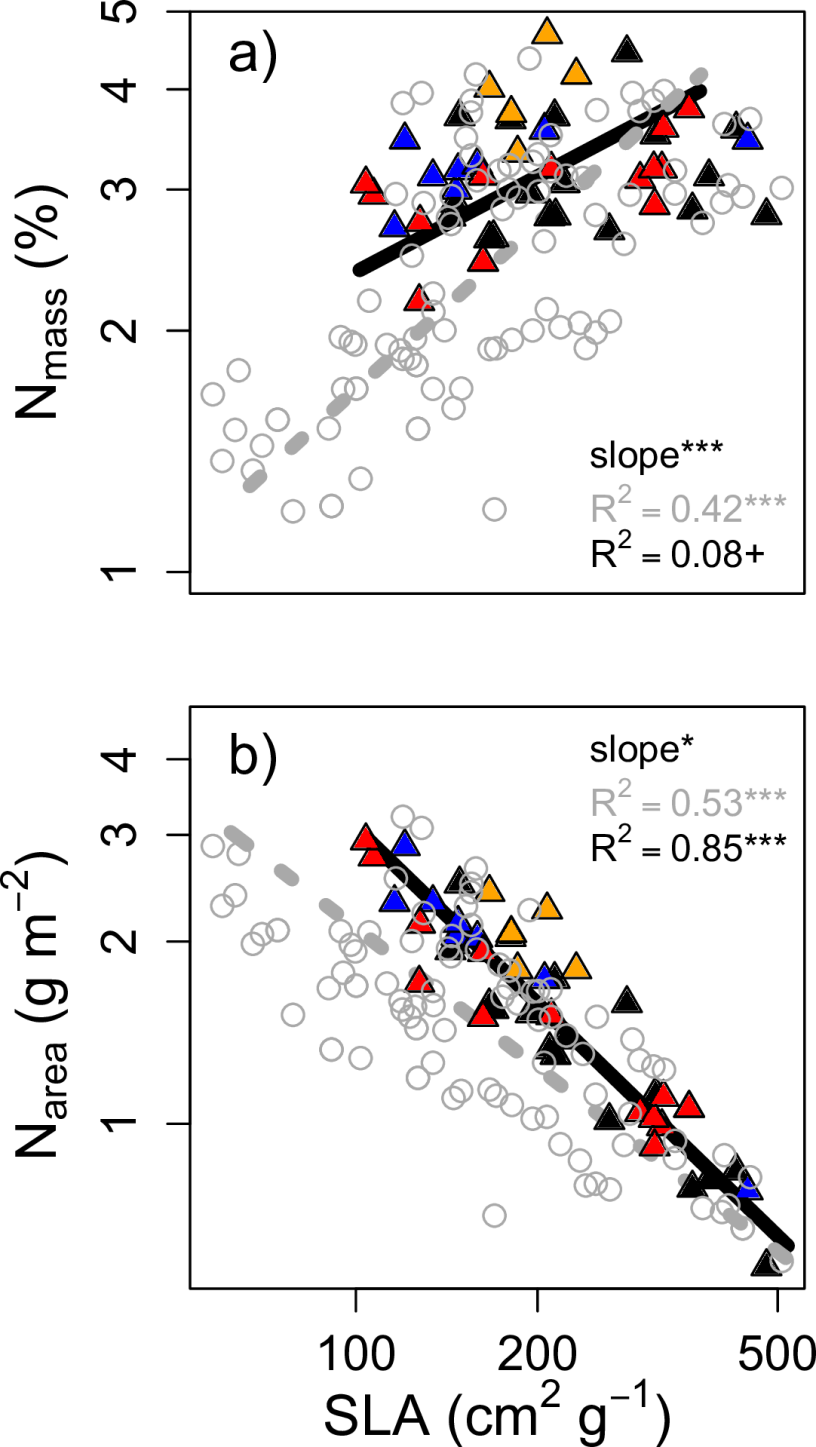
**Standardized major axis (SMA) relationships between (a) N**_**mass**_ **and SLA and (b) N**_**area**_ **and SLA.** Deciduous individuals are denoted by triangles (native closed, invasive open points) and native evergreen species (open circles) are shown in grey. +*P*<0.1; * *P*<0.05; ***P<0.001. Note axes are on log scale.

Considering intraspecific trait variation, most New Zealand endemic species deviated from invasive species in several trait relationships, especially when narrowing the comparison to only those dominant natives with an evergreen habit. In general, trait relationships were more pronounced when including all individuals, with most correlations being significant (*P*<0.05; Figs 3 and 4). These high trait correlations were partly due increased sample size compared to relationships across species means but also because of important intraspecific trait variation that was lost in analyses based on species means, particularly for N_mass_ (Fig 3F) and SLA (Fig 3H). Among mass-based relationships, correlations were always stronger for evergreen natives rather than deciduous species. Deciduous species exhibited reduced C gains per increase in respiratory costs (slope shift in A_max,mass_-R_d,mass_, Fig 3E), although the correlation was relatively low and completely absent on an area basis (Fig 4E). At a given leaf nitrogen investment (N_mass_, N_area_), deciduous species had consistently greater photosynthetic returns (A_max,mass_, A_max,area_), as indicated by significant elevation (y-intercept) shifts (Figs 3F and 4F). Although SLA did not differ significantly overall by leaf habit (Table 2), evergreen species occupied a greater range of SLA values. Per unit increase in SLA, evergreen species exhibited greater A_max,mass_ (slope shift, Fig 3H). Deciduous species tended occupy high SLA, high N_mass_ trait space, illustrated through a significantly lower slope in the N_mass_-SLA relationship (slope shift, *P*<0.001; Fig 5A). N_area_ was similar among evergreen and deciduous individuals, but, as with N_mass_, the tradeoffs (slopes) describing the relationship with SLA were also significantly different for deciduous and evergreen species (Fig 5B).

## Discussion

### Do endemic species follow unique resource-use strategies?

Our leaf-level analysis of native and invasive woody species in a New Zealand forest generally supports the broad hypothesis that species endemic to isolated regions exhibit distinct strategies (i.e., combination of trait values) compared to species that evolved in larger, more connected regions. These differences were largely modulated by functional differences in leaf habit. However, the degree to which our results represent ‘fundamental shifts’ in ecophysiological tradeoffs (significantly different slopes) was small. At the species level, invasive species occupied trait space associated with greater resource acquisition, with trait values shifted along a strategy axis towards higher A_max,mass_, N_mass_, SLA, and R_d,mass_. These shifts along a common trait scaling relationship are consistent with previous studies from across the globe [6,45]. In addition to higher photosynthetic rates than the majority of natives in the present study, we found invaders have higher nitrogen- and energy-use efficiencies, which also agrees with prior results in Eastern North American forests [9] and Hawaii [15]. Interestingly, the invaders in the present study, which were all deciduous, were similar to deciduous natives in nearly all traits and scaling relationships. Interspecific tradeoffs were constrained to broadly similar trait space and trend lines, in line with global analyses and conclusions for shared ecological strategies among species worldwide [4]. But, when including intraspecific variation, most New Zealand endemic species deviated from invasive species in several functional aspects, especially when narrowing the comparison to only those with an evergreen habit, a strategy which overwhelmingly dominates the native species pool.

Our results do not affirm our prediction that endemic species follow ‘fundamentally’ different carbon capture strategies. Alternatively, our data illustrate functionally distinct groups among species. As a group, invaders were shifted towards the ‘fast returns’ end of a shared leaf economics spectrum [4], characterized by high resource investments and high carbon returns. This interpretation largely echoes that of Leishman *et al*. [6], who found little evidence for differences in trait scalings among a large group of native and invasive species in Australia. Based on the absence of significant slope shifts across multiple sites, they concluded that native and invasive species shared similar carbon capture strategies. While the broad generality in trait relationships across species worldwide is well documented (e.g., [4]), we also stress the biological significance of deviations of species groups from a shared, general tradeoff surface [10,21]. Biophysical and genetic constraints play a key role in the evolution of plant form and function that dictate major axes of trait variation worldwide [5]. But understanding deviations from broad trends as a result of biogeographic constraints can provide important insights into diversity of evolved plant strategies, rather than simply disregarding heterogeneity as statistical noise [10]. It is important to note that some expected trait relationships were weakly or not correlated at all (e.g., A_area_-R_d,area_), despite high variation in nearly all leaf traits we measured. Of those tradeoffs that showed strong coordination, nativity differences in bivariate trait scaling (shifts in slope, y-intercept or along common slope) were subtle. Additionally, only three invasive species, none of which were evergreen, were measured. The coordinated variation in trait relationships demonstrated in global datasets (e.g., leaf economics spectrum) are limited by available trait variation at local scales, particularly leaf lifespan. Funk & Cornwell [17] argued that trait relationships are context-dependent, highlighting their finding that trait correlations were weak in communities dominated by species with low variation in leaf lifespan. In our analysis, we suspect differences between invaders and natives were confounded by strategies constrained by leaf habit. Therefore, additional species in habitats across New Zealand and with evergreen invaders are needed to fully assess the degree to which the native flora deviates from tradeoff surfaces generalized globally.

Previous research has highlighted differences between endemic and non-endemic species or comparing island natives to successful invaders. Gulias *et al*. [46] compared functional traits between Balearic endemic and non-endemic Mediterranean species and found endemic species were significantly different from more widespread species, with lower A_max,area_ and reduced slopes for A_max,mass_-N_mass_ and A_max,mass_-SLA relationships. These findings are similar in the present analysis, with 52% lower mean A_max,mass_ and lower A_mass,mass_ at a given N_mass_ for evergreen endemics compared to invasive species, but a greater slope for the A_max,mass_-SLA relationship. Likewise, several studies have compared native and invasive species in Hawaii, the most isolated floristic region of the world, to find significant differences in the slopes or of key trait tradeoffs, including greater slopes for invaders in A_max,area_-R_d,area_ [12] and A_max,mass_-N_mass_ [13].

Despite functional gaps in the New Zealand flora [24,27], native and invasive species measured at this site in the current study had similar traits and trait tradeoffs. Leaf area was the only trait that differed between native and invasive species. As a group, mean leaf area was 142% greater in invaders than natives (Table 2). This matches a common description of the distinctiveness of the endemic flora, noting small leaves and divaricating branching architectures [24]. The range in values for leaf area was large–from 0.85 cm^2^ in the endemic divaricating shrub *Melicope simplex* to 200 cm^2^ for the palmate leaves of the endemic tree *Pseudopanax arboreus*. Previous research has reported that leaf area in New Zealand canopy species are nearly an order of magnitude smaller compared to other temperate tree floras [25]. In the present study, the exact functional significance of large leaf area in invaders remains unknown, but we suspect these differences result both from of modern-day climatic and deeper time evolutionary constraints.

### Deciduousness as an invasive strategy?

Against our hypothesis, strategy differences were more a function of leaf habit rather than nativity. At this study site, native deciduous species (those with some degree of winter leaf loss) shared similar trait strategies as invaders. Unsurprisingly, deciduous and evergreen species have been shown to differ in certain traits, particularly SLA and leaf lifespan (e.g., [4]). The deciduous leaf habit has been associated with productive, resource-acquisitive plant strategies that are associated with high resource availabilities in seasonal climates [26]. Therefore, given that invasive species in general tend to possess such strategies [3], it makes sense that invaders in the present study are deciduous and share traits with native deciduous (or semi-deciduous) species. However, it is important to recognize that our results neglect leaf lifespan or whole plant carbon gain. Based on previous studies on similar sets of species in New Zealand [29,47,48], leaf lifespan is likely substantially shorter for the deciduous species we measured. It is unclear whether the deciduous trait strategies directly translate to fitness differences among species at this site. It is possible that whole plant C gain is similar between evergreen and deciduous species.

Deciduousness in New Zealand has long been a topic of interest, noting very few deciduous species [24,26,47,49]. McGlone *et al*. [26] summarized three leading hypotheses to explain New Zealand’s dearth of native deciduous species: 1) Phylogenetic constraint due to geographic isolation throughout the evolution of the flora, 2) weakly seasonal oceanic climate with mild winters, and 3) low soil fertility favors resource conservative and persistent strategies. They conclude mild New Zealand winters strongly support for the dominance of the evergreen habit. Further, small native deciduous trees are largely restricted to seral sites with greater nutrient availability than most New Zealand forest soils. Low soil fertility influences leaf lifespans [29], which likely provides disadvantages to deciduous species regionally. Additionally, the deciduous habit likely evolved *de novo* within New Zealand, which suggests phylogenetic constraint alone cannot explain the low numbers of deciduous species [26].

No native species in New Zealand are deciduous in the typical manner as temperate Northern Hemisphere species, where deciduous species rapidly produce a spring cohort of leaves, little to no leaf loss throughout the summer, and rapid leaf loss in autumn as days get shorter [26]. Bussell [47] compared three deciduous New Zealand species (two of which are included in present study) with *Acer pseudoplatanus* (an invasive species in the present study). Unlike endemic species, *A*. *pseudoplatanus* had clearly defined periods of bud burst and leaf fall. Further, *A*. *pseudoplatanus* is known to efficiently scavenge soil nitrogen in the native range, but with low leaf resorption rates prior to fall senescence [50]. It is unknown how this nutrient cycling strategy compares to New Zealand deciduous species. This lack of a “classic” deciduous habit among native species might contribute to phenotypic novelty and success of many invaders in New Zealand, but is probably not the sole reason (e.g., enemy release, propagule pressure, anthropogenic disturbance). Interestingly, this invasion pattern is in stark contrast to that reported for Eastern North American forest invasions, where woody invaders tend to have an extended phenology into autumn and longer leaf lifespans than natives [9,51].

Assuming deciduousness is indeed advantageous at our study site, it is puzzling why the native deciduous species we measured are not more locally abundant. Despite being labelled as deciduous, it is likely that native deciduous species in New Zealand are phenologically and functionally different than deciduous species in the Northern Hemisphere. Comparisons between deciduous and evergreen species tend to be focused on species with very different leaf longevities (e.g., deciduous trees vs. conifers in the Northern Hemisphere), but deciduousness can vary from complete loss of leaves prior to winter to only partial leaf loss over winter to leaf drop and flushes at the onset of spring [26]. Substantial differences in leaf phenology and annual carbon gain has been shown between winter deciduous *Fuchsia excorticata* and semideciduous *Aristotelia serrata* [48], both of which were included as native deciduous species in the present study. In the present study, we did not measure leaf lifespan or canopy phenology, which could further explain interspecific strategy differences or invasion success. Native species have been shown to vary considerably in leaf habit and those categorized as “deciduous” in the current analysis do not necessarily share leaf habit [26]. For example, *P*. *regius* is the only measured species that is deciduous throughout its range, while *F*. *excorticata* exhibits complete winter leaf loss (but with evergreen populations in the northern New Zealand). Similarly, *A*. *serrata* exhibits partial winter leaf loss, while *S*. *microphylla* and *H*. *angustifolia* can show outer canopy winter leaf loss. Secondly, although we did not measure leaf phenology at this site, leaf lifespans for this suite of native evergreen species varies from barely exceeding one year (annual evergreens) to those well exceeding one year. Lacking both site specific phenological data for this and the sample size to reasonably test for differences between “true deciduous,”, “semideciduous,” “brevideciduous,” “short/annual evergreen”, and “multiannual evergreen” groups, we limited our current analysis to the binary categories of species with significant leaf loss (deciduous) compared to those with marked leaf loss in winter. Lastly, our conclusions were limited to one site with data for three invasive species, none of which were evergreen. Future work is needed to expand native-invasive trait comparisons to additional sites in order to capture diversity of trait strategies of the native and exotic flora of New Zealand.

## Conclusions

Our results provide a traits-based perspective for successful invasion into a functionally depauperate flora. From a leaf-level perspective, we found invasive species, all of which were deciduous, to have similar trait strategies as native deciduous species. Deciduous species in this predominately evergreen forest exhibited both higher productive traits and more efficient resource use than the endemic evergreen species. Leaf trait associations were in similar directions among species groups, which in a broad sense, indicates shared physiological tradeoffs between endemic and invasive species. However, we found significant deviations in common leaf economic tradeoffs when considering within species variation. Additional work is needed on the physiology of the various forms of the deciduous habit in New Zealand compared to elsewhere. Further, the invasion of deciduous species into predominately evergreen communities could produce significant shifts in biogeochemical cycles.

## Supporting information

S1 Table Model parameters and prior distributions used in the photosynthetic light response model. (DOCX)

S2 Table Summary of leaf trait data for each species. (XLSX)

S1 File Description of the hierarchical Bayesian photosynthetic light response model. (DOCX)

S2 File R code for Bayesian photosynthetic light response model. (DOCX)
