# Supplementary material for: Are endemics functionally distinct? Leaf traits of native and exotic woody species in a New Zealand forest
Source: PLoS One. 2018 May 2;13(5):e0196746. doi: 10.1371/journal.pone.0196746 (PMC5931660; doi:10.1371/journal.pone.0196746)
Supplement: S1 Table — (DOCX) [file pone.0196746.s001.docx]

**Table S1.** Model parameters and prior distributions used in the light response curve models. All prior distributions were non-informative.

| **Symbol** | **Definition (units)** | **Attribute** | **Distribution**  **(mean μ, sd σ)** |
| --- | --- | --- | --- |
| A_net_ | Net photosynthetic rate (observed)  (µmol CO_2_ m^-2^ s^-1^) | Dependent variable | Data |
| μA_net_ | Net photosynthetic rate (modeled)  (µmol CO_2_ m^-2^ s^-1^) | Dependent variable | Predicted value |
| τ | Model precision (variance^-1^) | Parameter | (model σ^2^)^-1^  σ ~ dunif(0,100) |
| R_d_ | Dark respiration rate (µmol CO_2_ m^-2^ s^-1^) | Parameter | dnorm(µ=0, σ^2^=10^3^) |
|  | Apparent quantum yield (µmol CO_2_ m^-2^ s^-1^) | Parameter | dnorm(µ=0.054, σ^2^=10^5^)>0 |
| θ | Curvature parameter (dimensionless) | Parameter | dnorm(µ=0.5, σ^2^=10^3^) >0 and <1 |
| A_max_ | Maximum gross photosynthetic rate  (µmol CO_2_ m^-2^ s^-1^) | Parameter | dnorm(µ=0, σ^2^=10^5^) |
| RE_p,s_,  RE_p,i_ | Random individual effects for species, *s*, or individual, *i*, on parameter, *p* | Parameter | (RE σ^2^)^-1^  σ ~ dunif(0,100) |
| PPFD | Photosynthetic photon flux density (µmol photons m^-2^ s^-1^) | Independent variable | - |
|  |  |  |  |
