# Supplementary material for: Are endemics functionally distinct? Leaf traits of native and exotic woody species in a New Zealand forest
Source: PLoS One. 2018 May 2;13(5):e0196746. doi: 10.1371/journal.pone.0196746 (PMC5931660; doi:10.1371/journal.pone.0196746)
Supplement: S1 File — (DOCX) [file pone.0196746.s003.docx]

**File S1.** Description of the hierarchical Bayesian photosynthetic light response model

*Model structure*

Our likelihood function for photosynthetic data is based on the likelihood of each observation, (A_net,_*_i_*), from *i* – N, assumed to follow a normal distribution:

$A_{net,i} \sim Normal(\mu A_{net,i},\tau)$ (S1.1)

where μA_net_ is the predicted photosynthetic rate and τ is precision (1/variance). Our process model describing the photosynthetic light response:

${\mu A}_{net}=\frac{ɸPPFD+A_{max}-\sqrt{\left( \phi PPFD+A_{max} \right)^{2}-4ϴ\phi PPFD\left( A_{max} \right)}}{2ϴ}-R_{d}$ (S1.2)

where A_net_ and A_max_ are the area-based net and maximum gross photosynthetic rates (μmol CO_2_ m^-2^ s^-1^), respectively, is the apparent quantum yield (mol CO_2_ mol photons^-1^), R_d_ is daytime dark respiration rate (|A_net_| at no light; μmol CO_2_ m^-2^ s^-1^), and θ is curve convexity (dimensionless). Apparent quantum yield () and dark respiration ($R_{d}$) were jointly from a simple regression of Anet-PPFD under low light levels:

${\mu A}_{net,}={{PPFD}_{low}-R}_{d}$ (S1.3)

where PPFD_low_ includes light levels <100 μmol photons m^-2^ s^-1^.

Species and individual-level random effects were included as follows:

$$Parameter= Parameter'+{RE}_{parameter,sp}+ {RE}_{parameter,ind}$$

where $Parameter$ includes the main model parameters (except θ), $Parameter'$ is the overall intercept, *RE_parameter,,sp_* and *RE_parameter,ind_* are the random effects of species and individuals, respectively. First, separate models were run for each nativity x leaf habit group to extract group-level estimates. Second, a species-level model included random effects only (Fig. 1,2). Last, the full model included species and individual random effects (Fig. 3, 4).
