# Supplementary material for: Are endemics functionally distinct? Leaf traits of native and exotic woody species in a New Zealand forest
Source: PLoS One. 2018 May 2;13(5):e0196746. doi: 10.1371/journal.pone.0196746 (PMC5931660; doi:10.1371/journal.pone.0196746)
Supplement: S2 File — (DOCX) [file pone.0196746.s004.docx]

**File S2.** R code for Bayesian photosynthetic light response model, fit via JAGS using the R2jags library.

#load R2jags package installed in R

library(R2jags)

#Inputs include:

spnumber #vector of coded species; 1-18

N.spp = 18 #number of species

indiv #vector of coded individuals; 1-93

N.indiv = #number of individuals

N = 1063 #total gas exchange observations

q #light levels; PPFD

Anet #observed photosynthetic rates

qlrc #light levels for first part of curve (q<100)

Anetlrc #observed photosynthetic rates for first part of curve (q<100 data subset)

N.LRC = 275 #number of gas exchange observations (q<100 data subset)

indivLRC #coded individuals (q<100 data subset)

sppnumberLRC = #number of species (q<100 data subset)

N.indivLRC = #number of individuals (q<100 data subset)

subset_AQY #vector of row numbers to match individuals from q<100 data subset to full curve

#leaf traits used for estimating parameters on mass basis and resource use efficiency metrics

#(trait data available in Table S2)

SLA #specific leaf area

LeafN #leaf nitrogen

Trans #leaf transpiration rate at saturating light

CC #leaf construction cost

#=============================

#model specified in BUGS syntax

#=============================

mod <- "model

{

#Nonrectangular hyperbola light response curve model

#non-informative priors

Amax.int ~ dnorm(0,0.00001) I(0, ) #Amax intercept; bounded by zero

theta.int ~ dnorm(0.5,0.001) I(0,1) # theta intercept; bounded by 0,1

Rd.int ~ dnorm(0,0.001) #dark respiration intercept

AQY.int ~ dnorm(0.054,0.001) I(0, ) #apparent quantum yield; bounded by zero

tau <- sigma^-2 #convert SD to precision (1/variance)

sigma ~ dunif(0, 100) #uniform prior

spp.tau.Amax <- spp.sigma.Amax^-2 #convert SD to precision (1/variance)

spp.sigma.Amax ~ dunif(0, 100) #uniform prior for SD

ind.tau.Amax <- spp.sigma.Amax^-2 #convert ind SD to precision (1/variance)

ind.sigma.Amax ~ dunif(0, 100) #uniform prior for SD

for(i in 1:N.LRC) { #loop through low light observations only

vc[i] ~ dnorm(mu[i],tau.AQY[i])

mu[i] <- b0[i] + b1[i]*qlrc[i]

b1[i]<- AQY.int + b.spp.AQY[sppnumberLRC[i]] + b.ind.AQY[indivLRC[i]]

b0[i]<-Rd.int + b.spp.Rd[sppnumberLRC[i]] + b.ind.Rd[indivLRC[i]]

tau.AQY[i] <- 1 / sqrt(sigma.AQY^2)

}

#non-informative priors for first part of curve fit (AQY)

sigma.AQY ~ dunif(0,100)

tau.spp.AQY <- sigma.spp.AQY^-2

sigma.spp.AQY ~ dunif(0,100)

tau.spp.Rd <- sigma.spp.Rd^-2

sigma.spp.Rd ~ dunif(0,100)

tau.ind.AQY <- sigma.ind.AQY^-2

sigma.ind.AQY ~ dunif(0,100)

tau.ind.Rd <- sigma.ind.Rd^-2

sigma.ind.Rd ~ dunif(0,100)

#random AQY intercept for species

for(i in 1:N.spp) {

b.spp.AQY[i] ~ dnorm(0,tau.spp.AQY)}

#random Rd intercept for species

for(i in 1:N.spp) {

b.spp.Rd[i] ~ dnorm(0,tau.spp.Rd)}

#random AQY intercept for individuals

for(i in 1:N.indivLRC) {

b.ind.AQY[i] ~ dnorm(0,tau.spp.AQY)}

#random Rd intercept for individuals

for(i in 1:N.indivLRC) {

b.ind.Rd[i] ~ dnorm(0,tau.ind.Rd)}

for(i in 1:N) { #loop through all observations

Anet[i] ~ dnorm(An[i],tau) #Anet is the observed response, which takes on the predicted value An plus normal error

An[i] <- ((1/(2*theta[i]))*(AQY[i]*q[i]+Amax[i]-((AQY[i]*q[i]+Amax[i])^2-4*AQY[i]*theta[i]*Amax[i]*q[i])^(1/2))-Rd[i])

theta[i]<-theta.int

AQY[i]<-b1[subset_AQY[indiv[i]]]

Rd[i]<- abs(b0[subset_AQY[indiv[i]]])

Amax[i]<-Amax.int + b.spp.Amax[spnumber[i]]+ b.ind.Amax[indiv[i]]

Amass[i] <- Amax[i]*SLA[i]*(1/10000)

Rdmass[i] <- Rd[i]*SLA[i]*(1/10000)

A.R[i] <- Amax[i]/Rd[i]

LCPT[i] <- Rd[i]/AQY[i]

PNUE[i] <- Amax[i]/leafN[i]

WUE[i] <- Amax[i]/Trans[i]

PEUE[i] <- Amass[i]/CC[i]*(0.1)

} #end loop

#random intercept for species (A/q)

for(i in 1:N.spp) {

b.spp.Amax[i] ~ dnorm(0,spp.tau.Amax)

}

#random intercept for individual (A/q)

for(i in 1:N.indiv) {

b.ind.Amax[i] ~ dnorm(0,spp.tau.Amax)

}

}" #end model

#Write model to text file for JAGS

write(mod, "model.txt")

#input lists for JAGS

params = c("Amax.int","theta.int","AQY.int","sigma","sigma.AQY","LCPT","Amax","Rd.int", "AQY", "Rd","Amass","Rdmass","A.R","PNUE","WUE","PEUE","Amass.avg","Rdmass.avg","A.R.avg","LCPT.avg","PNUE.avg","WUE.avg","PEUE.avg") #parameters to monitor

inits = function() list(Amax.int=rnorm(1,mean=8,sd=1),theta.int=abs(rnorm(1,mean=0.5,sd=0.1)),Rd.int=rnorm(1,mean=0,sd=1),AQY.int=abs(rnorm(1,mean=0.05,sd=0.05))) #starting values for fixed effect parameters

input = list(N=N,q=q,Anet=Anet,vc=Anetlrc,qlrc=qlrc,N.LRC=N.LRC,subset_AQY=subset_AQY,leafN=leafN,SLA=SLA,CC=CC,Trans=Trans,N.indiv=N.indiv,indiv=indiv,N.indivLRC=N.indivLRC,indivLRC=indivLRC,SLA=SLA,leafN=leafN,Trans=Trans,CC=CC) #input

#seed random seed (not required but recommended for repeat runs)

set.seed(rnbinom(1, mu=200, size=1))

#run JAGS model

Model.NZ <- jags(model = "model.txt",data = input,inits=inits,param=params,

n.chains = 3, #number of separate MCMC chains

n.iter =100000, #number of iterations per chain

n.burnin = 50000) #number of initial iterations to discard

#eventually increase iterations and burn in; 20000,5000burnin to start
